# Physical and Chemical Activation of Graphene-Derived Porous Nanomaterials for Post-Combustion Carbon Dioxide Capture

**DOI:** 10.3390/nano11092419

**Published:** 2021-09-17

**Authors:** Rabita Mohd Firdaus, Alexandre Desforges, Mélanie Emo, Abdul Rahman Mohamed, Brigitte Vigolo

**Affiliations:** 1 School of Chemical Engineering, Engineering Campus, Universiti Sains Malaysia, Nibong Tebal, Seberang Perai 14300, Penang, Malaysia; rabitafirdaus58@student.usm.my; 2 Université de Lorraine, CNRS, IJL, F-54000 Nancy, France; Alexandre.Desforges@univ-lorraine.fr (A.D.); Melanie.Emo@univ-lorraine.fr (M.E.)

**Keywords:** graphene oxide, activation, porosity, adsorption, carbon dioxide

## Abstract

Activation is commonly used to improve the surface and porosity of different kinds of carbon nanomaterials: activated carbon, carbon nanotubes, graphene, and carbon black. In this study, both physical and chemical activations are applied to graphene oxide by using CO_2_ and KOH-based approaches, respectively. The structural and the chemical properties of the prepared activated graphene are deeply characterized by means of scanning electron microscopy, Raman spectroscopy, Fourier transform infrared spectroscopy, X-ray photoelectron spectrometry and nitrogen adsorption. Temperature activation is shown to be a key parameter leading to enhanced CO_2_ adsorption capacity of the graphene oxide-based materials. The specific surface area is increased from 219.3 m^2^ g^−1^ for starting graphene oxide to 762.5 and 1060.5 m^2^ g^−1^ after physical and chemical activation, respectively. The performance of CO_2_ adsorption is gradually enhanced with the activation temperature for both approaches: for the best performances of a factor of 6.5 and 9 for physical and chemical activation, respectively. The measured CO_2_ capacities are of 27.2 mg g^−1^ and 38.9 mg g^−1^ for the physically and chemically activated graphene, respectively, at 25 °C and 1 bar.

## 1. Introduction

Emissions of carbon dioxide (CO_2_) from manufacturing plants and automobiles have caused severe environmental concerns. It has been widely accepted that the accumulation of greenhouse gases in the atmosphere causes an enhanced greenhouse effect, thus resulting in global warming and climate change [1]. The gradual rise in atmospheric CO_2_ from human activities and industry effluents necessitates research aimed toward carbon capture [2]. Liquid-based absorption technology has many limitations, including toxic by-products and high energy demand for the adsorbent regeneration; thus, research is underway to create a more reliable approach for absorbing CO_2_ in post-combustion environments. To date porous carbon materials including graphene offer a wide variety of chemical composition and structural architectures that are suitable for the adsorption and storage of different gas molecules including hydrogen [3], methane [4] and carbon dioxide [5]. In addition, these materials also display great promise in terms of the surface functional groups, for example, the self-organization, chemical stability, reactivity, etc., in adsorptive processes. Besides surface chemical changes, the pore volume and pore size distribution are the crucial keys for material properties studied and modified to improve CO_2_ adsorption ability. However, several variables must be considered, and determining the function of each of them is challenging.

A great number of investigations have been reported demonstrating the role of surface chemistry of carbon materials, such as graphene, on CO_2_ adsorption [5,6,7,8]. Relatively simple and numerous methods including physical or chemical activation are currently known for the modification of the surface chemistry of graphene along with the deep influence that it exerts in the properties of the resulting materials [9,10]. In general, physical activation is performed at high temperature (>700 °C) in a CO_2_ or steam atmosphere, and it consists of oxidation of carbon-based products under a stream of an oxidizing gas such as water vapor, oxygen, and carbon dioxide [11,12]. CO_2_ is often favored because of its lower reactivity at high temperatures, rendering the activation process easier to regulate. Furthermore, CO_2_ activation has been shown to promote microporosity development in the early phases of activation, whereas steam rather favors microporosity widening, thus activated materials produced with steam have a smaller micropore volume [13]. For chemical activation, the process usually has several steps, including one thermal step combined with several chemical approaches. Different activating agents have been traditionally studied in the literature, such as zinc chloride (ZnCl_2_) [14], phosphoric acid (H_3_PO_4_) [15], sodium hydroxide (NaOH) [16] and potassium hydroxide (KOH) [17,18,19,20]. NaOH and KOH appear to be the most preferred activation agents among the findings. However, depending on the type of the material used, each of these two activation agents are expected to work differently. For example, Raymundo-Pinero et al. stated that, despite the fact that NaOH is considered as an efficient activating agent, it cannot activate most forms of carbon nanotubes, which is consistent with the results obtained with other types of carbonaceous precursors, particularly graphene [21]. The purpose of activation is to improve the specific surface area or pore volume of activated graphene through opening new pores and developing the existing pores. Additionally, activation can vary or adjust the surface chemical nature of activated graphene with certain unique characteristics. Since the physicochemical properties of carbons are highly affected by the existence of chemical compounds on the surface, and their chemical characteristics, understanding the surface chemistry of activated graphene is critical in adsorption study. In this study, both physical and chemical activation approaches have been applied to graphene oxide (GO) as starting material and the activated GOs were used for CO_2_ adsorption. CO_2_ and KOH-based activation methods have been used as physical and chemical activation, respectively. The structural and the chemical properties of the prepared activated GOs have been deeply characterized by means of complementarily techniques: scanning electron microscopy (SEM), transmission electron microscopy (TEM), Raman spectroscopy, Fourier transform infrared spectroscopy (FTIR), X-ray photoelectron spectrometry (XPS) and nitrogen adsorption. The impact of the used temperature for the activation treatment on the CO_2_ adsorption capacity has been investigated and discussed by means of a proposed mechanism.

## 2. Materials and Methods

### 2.1. Materials and Chemicals

Graphite powder (purity 99.9995%), sulphuric acid (H_2_SO_4_, 95–97%), sodium nitrate (NaNO_3_), potassium permanganate (KMnO_4_) and KOH were purchased from Sigma-Aldrich. Hydrogen peroxide (H_2_O_2_, 30%) and hydrochloric acid (HCl, 37%) were purchased from Chem-Supply and Biolab respectively. CO_2_ and N_2_ gases were supplied by Azeratech Engineering (Pulau Pinang, Malaysia).

### 2.2. Synthesis of GO

The Hummers’ method was used to synthesize GO from graphite powder [22]. The typical procedure is as follows: graphite (2 g) and NaNO_3_ (2 g) were mixed with H_2_SO_4_ (50 mL) and stirred for 2 h in an ice bath (0–5 °C). Then, 6 g of KMnO_4_ was slowly added to the resulting solution. The ice bath was removed, and the solution was then stirred at 35 °C for 2 days. An amount of 300 mL of deionized water (DI) was added gently to the solution. Then, 10 mL of H_2_O_2_ were slowly added to the above solution. The resulting solution was washed with HCl (400 mL, 10%). Additional washing with DI water afforded the GO product as a powder. Subsequently, the GO powder samples were dried using a freeze dryer.

### 2.3. Physical Activation of GO

GO was activated in a simple one-step reaction using CO_2_ as the activation agent. In a typical procedure, 0.5 g of the synthesized GO was loaded on a ceramic boat, placed at the center of the tube furnace, and heated under a continuous flow of CO_2_ (100 mL min^−1^) to the desired temperature (500, 600, 700, 800 and 900 °C) at a heating rate of 10 °C min^−1^ and a holding time of 1 h. Following the activation step, the sample was left to cool naturally to room temperature in the furnace under the flow of N_2_. The materials thus obtained were labelled as GO-PA X, where X is the activation temperature.

### 2.4. Chemical Activation of GO

An amount of 0.5 g of GO was soaked in an aqueous KOH solution (1.5 g in 1 mL) using a ratio of 1:3. After 6 h of contact time, the mixture was dried at 110 °C for 24 h. Later, each sample was loaded into the horizontal tube furnace under a flowing N_2_ atmosphere (100 mL min^−1^) and was heated at 10 °C min^−1^ to 500 °C. Once the maximum temperature was reached, a reaction time of 60 min was applied. After the heat treatment, the samples were washed with HCl (1 M), then washed with distilled water until pH 6. This procedure was repeated using a different range of temperature in the 600–800 °C range. The materials thus obtained were labelled as GO-CA X, where X is the activation temperature.

### 2.5. Material Characterization

Several characterization techniques were employed in this study to characterize the physicochemical properties of the produced adsorbent samples. Scanning electron microscopy (SEM) was carried out using a FEI Quanta 650 FEG microscope (FEI, Dreieich, Germany) operated at 10 and 15 kV to observe the morphology of the samples. TEM observations were performed using a JEOL JEM-ARM 200F apparatus at 80 kV (Jeol Europe, Croissy-sur-Seine, France). Fourier transform infrared (FTIR) analysis was conducted using a Thermo Scientific Nicolet iS10 spectrometer with KBr pellet (ThermoFisher Scientific, Hillsboro, OR, USA). To gain some insight into the intrinsic carbon structure of the prepared samples, a Renishaw in Via Raman microscope (Renishaw plc, Wotton-under-Edge, UK) was used with an excitation wavelength of 632.8 nm, a laser power output of 50 mW focused on the sample with an x50 objective lens. For each sample, at least 3 spectra were recorded on different areas of the sample deposited on a glass slide. For data analysis, after subtracting a baseline, the intensity of the D or the G band corresponding to the height at the maximum intensity of the peak was used to calculate the intensity ratio I_D_/I_G_. The surface area and pore size were evaluated by Brunauer–Emmett–Teller (BET) (Micromeritics Ltd, Dunstable, UK) standard via nitrogen adsorption at 77 K using a Micromeritics ASAP 2020 V4 volumetric adsorption apparatus and Barrett–Johner–Halenda (BJH) method, respectively, using an Autosorb 1C Quantachrome analyzer. The BET surface area is also known as SSA. X-ray photoelectron spectroscopy (XPS) analysis was also conducted to determine the chemical state and concentration of elements on the adsorbent surface using an ULVAC-PHI Quantera II XPS equipped with an Al Kα X-ray source (hv = 1486.6 eV) (ULVAX-PHI, Inc., Kanagawa, Japan).

### 2.6. CO_2_ Adsorption Study

The CO_2_ adsorption performance of the developed adsorbent samples was assessed isothermally in a Thermogravimetric Analyzer (TGA, SDTQ-600, TA Instruments, Eschborn, Germany). For this purpose, around 15 mg of the adsorbent was loaded in the TGA pan and heated at the heating rate of 20 °C min^− 1^ to 110 °C under N_2_ flow of 75 mL min^−1^. The sample was kept isothermally at 110 °C for 15 min purposely to remove all the moisture or any pre-adsorbed CO_2_ from the sample. The temperature was then equilibrated to temperature 30 °C to prepare for the CO_2_ adsorption process. A CO_2_ flow of 75 mL min^−1^ was introduced to initiate the CO_2_ adsorption and held at this adsorption temperature for 90 min. The weight changes indicated the amount of CO_2_ captured were continuously recorded for further calculation of CO_2_ adsorption capacity (mg g^−1^). CO_2_ adsorption capacity was referred to the amount of CO_2_ gas adsorbed by the adsorbent at a specific time (duration of the CO_2_ adsorption test) and was calculated.

## 3. Results

### 3.1. Structural and Chemical Modifications of GO through Activation

**Figure 1:**
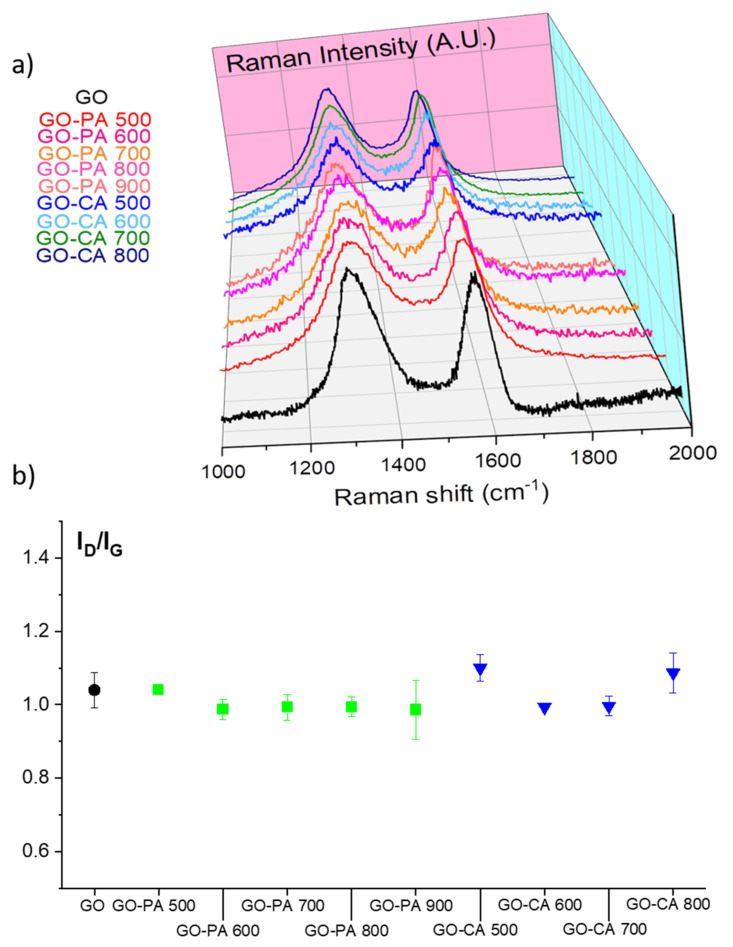
(**a**) Raman spectra and (**b**) corresponding I_D_/I_G_ of pristine GO and the activation GO by both the chemical and the physical activation methods.

Raman spectroscopy is a widely used technique for carbon nanomaterials since it allows researchers to investigate the structural modifications of the carbon network subsequently to chemical treatments. Raman spectroscopy was carried out on the prepared GO samples to follow the effect of activation temperature on the structural properties of GO. The two chief peaks observed for all spectra around 1370 and 1620 cm^−1^ represent the amorphous (D-band) and crystalline (G-band) carbon structures, respectively, (Figure 1a). The intensity of the D band (with respect to that of the G band) is known to increase when defects are introduced within the sp^2^ carbon network. The larger the degree of defect, the lower the density ratio of G band to D band (I_G_/I_D_) [23]. The calculated I_D_/I_G_ for the starting GO and all the treated GO samples are shown in Figure 1b.

The pristine GO sample possessed high I_D_/I_G_ ratio (1.04 ± 0.05), indicating the presence of defects/functional groups due to the strong oxidative conditions during its synthesis, as expected. After both activation treatment, the I_D_/I_G_ ratio remain around 1, meaning that, in average, activation does not impact on the structural quality of the activated GOs. The nature of the functional groups were next studied by FTIR for all the activated GO samples.

**Figure 2:**
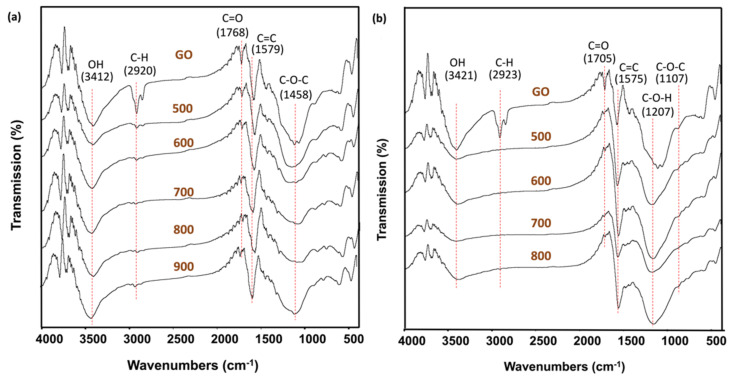
FTIR spectra of (**a**) physical activation and (**b**) chemical activation of GO at different temperatures.

Basically, FTIR allows researchers to study the effect of thermal/chemical history on the eventual nature changes of the grated functional groups. The FTIR spectra of GO and activated GOs by chemical and physical activation are illustrated in Figure 2a,b, respectively. The strong peak located at about 1575–1579 cm^−1^ is the signature of the skeletal C=C vibrations in the aromatic ring structure, as expected for GO. The wide band at around 3400 cm^−1^ is attributed to the O-H stretching vibrations of hydroxyl or carboxyl groups. The bonds associated with oxygen-containing groups in GO were considerably decreased and some of these bonds disappeared after GO was activated. As compared to physical activated and pristine graphene, the O-H peak for chemically activated GO is less intense, which might suggest that it was undergoing a strong reduction process. The band appearing at around 2900 cm^−1^ corresponds to sp^3^ C–H stretching vibration in aliphatic and aromatic structure. The sharp unsymmetrical peak at 1744 cm^−1^ of GO may be attributed to the C=O stretching vibrations. With an increase in activation temperature for both chemical and physical activation, the features related to carbon-oxygen bonds become less intense. Each activation approaches obviously lead to an effective reduction of GO [23].

### 3.2. Optimized Activation Conditions to Enhance CO_2_ Adsorption

**Figure 3:**
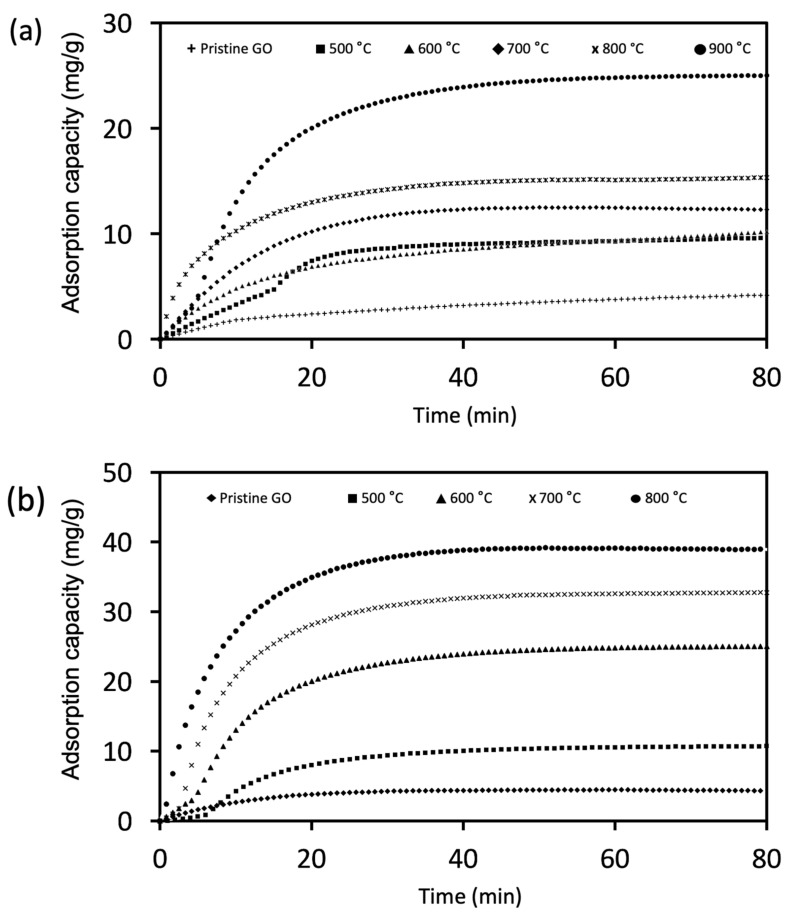
CO_2_ adsorption isotherms of GO after (**a**) physical activation and (**b**) chemical activation at different temperatures.

The impact of both chemical and physical activation approaches conducted at different temperatures to prepare graphene-based adsorbents for CO_2_ capture were examined. In this case, similar adsorption trends were achieved for both physically and chemically activated GO. The adsorption capacity increases when the activation temperature for physical and chemical increases from 500 to 900 °C and from 500 to 800 °C, respectively, as shown in Figure 3a,b. According to the findings, it shows that physical activation improves the adsorption capacity of pristine GO by a factor of 6.5, from 4.2 mg g^−1^ to 27.2 mg g^−1^, whereas chemical activation boosts the adsorption capacity by a factor of 9 from 4.3 mg g^−1^ to 38.9 mg g^−1^ (Table 1).

**Figure 4:**
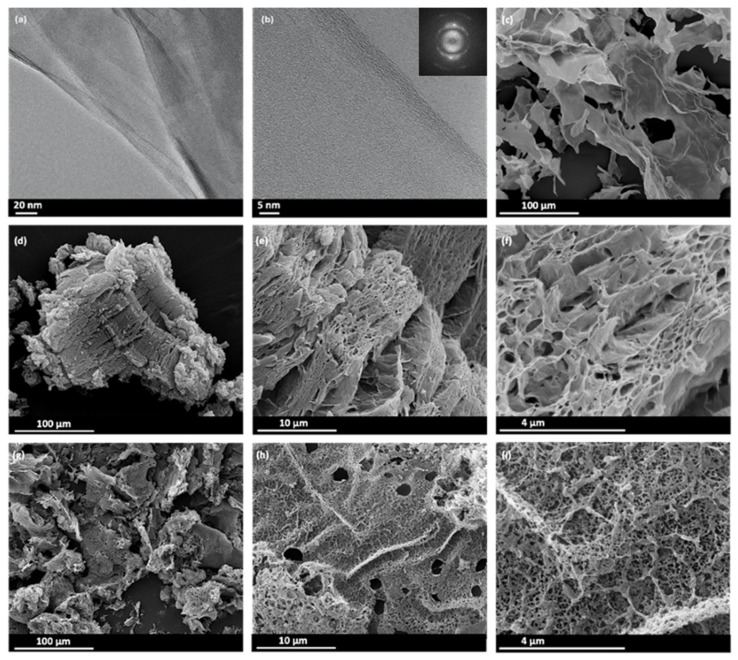
TEM images of GO (**a**) low-magnification (**b**) high-magnification, insert: corresponding FFT; (**c**) SEM image of GO; SEM images of GO-PA 900 at (**d**) low-magnification (**e**-**f**) high-magnification; SEM image of GO-CA 800 (**g**) low-magnification (**h**-**i**) high-magnification.

In the following, an in-depth characterization of three samples GO, GO-PA 900 and GO-CA 800, with the two latter showing the best adsorption capacity, has been performed. The morphology of all the samples was observed using TEM and SEM. The TEM images of GO at different magnifications are shown on Figure 4a,b. GO consists of less than 10 graphene layers stacked together in thin nanosheets. The spacing distances d_hkl_ obtained from the Fast Fourier Transform (FFT) patterns (insert Figure 4b) correspond to a graphite structure (P6_3_/mmc, d_100_ = 2.090 Å, d_110_ = 1.206 Å). The interspacing of the GO layers of 0.37–0.4 nm is as well consistent with a typical GO structure (Figure S1, Supplementary Materials).

SEM was used to study the microstructure and morphology of the starting material (GO), GO-PA 900 and GO-CA 800. The typical thin sheets of GO are well visible by SEM (Figure 4c) in agreement with TEM (Figure 4a,b). The light and porous structure of both GO-PA 900 and GO-CA 800 is evidenced by SEM. The meso-macropores of the GO-PA 900 with a diameter ranging from several μm to larger than 50 μm is observed in Figure 4d–f. The macroporosity of the GO-PA 900 remains intact even after CO_2_ activation.

**Figure 5:**
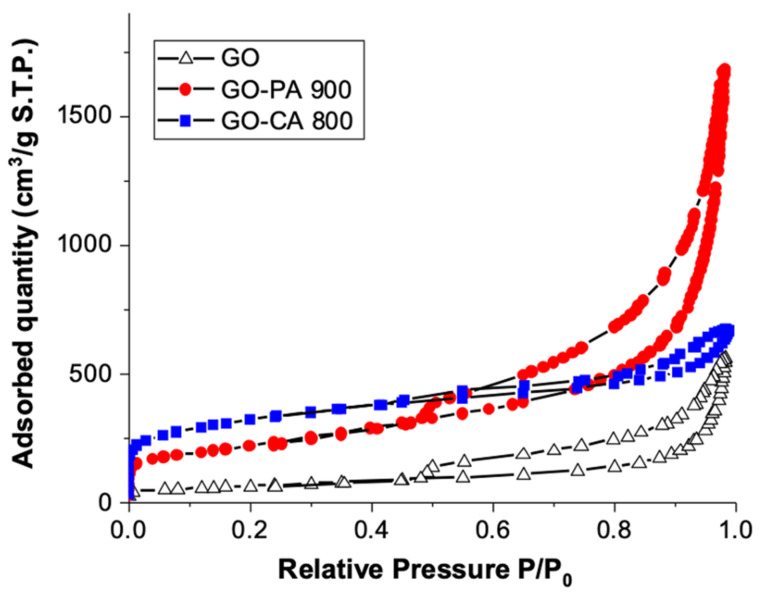
N_2_ adsorption-desorption isotherms of the pristine GO, GO-PA 900 and GO-CA 800.

Surface area analysis was performed on the pristine GO and activated GO samples. Figure 5 shows nitrogen adsorption-desorption isotherms of all the samples. GO and GO-PA 900 indicate Type IV isotherm, as defined by IUPAC classification showing the presence of mesopores [24]. Meso and micropores were seen in sample GO-CA 800 as it exhibited both Type I and Type IV isotherms. The mesoporosity in the sample is due to the aid of activating agents at high temperature. The isotherm of all the samples indicates H3 hysteresis loop. The hysteresis loop starts at a relative pressure range of 0.5–1 which indicates capillary condensation phenomena, characteristic of mesoporous materials [25]. Hysteresis is basically a condition where the adsorption and desorption curves do not overlap, associated with the capillary condensation phenomenon that takes place in mesoporous structures during desorption process [26]. In this case, narrow hysteresis loops for the activated GO were observed, which signifies the less formation of mesopores compared to pristine GO. The microporosity fraction of activated GOs is indeed around 13.19 % compared to 1.02 % in pristine GO (Table 2). The appearance of H4 hysteresis loop was clearly spotted in sample GO-CA 800, indicating the existence of micro-mesopores structure.

The quantitative results from adsorption analysis are shown in Table 2. The results reveal that GO has the lowest SSA of 219.32 m^2^ g^−1^, whereas KOH activation at 800 °C appears to be the most effective in increasing surface area and porosity with the highest SSA (1060.5 m^2^ g^−1^), followed by CO_2_ activation at 900 °C (762.5 m^2^ g^−1^). Furthermore, the pore size decreases significantly after chemical activation, from 13.67 nm to 3.65 nm and in a lower extent to 12.14 nm after physical activation. Since CO_2_ has a relatively small kinetic diameter of 0.33 nm, it is widely known that microscopic pores are required for its efficient gas molecule diffusivity into the adsorbent framework [27]. Similarly, Ma et al. found that after activating graphene oxide sheets with KOH, the SSA of activated graphene increased by a factor of 3.7, along with the formation of micropore structure, as compared to pristine graphene [28]. Wu et al. discovered that as well that at higher activation temperatures, micropores were fully developed [29]. Theoretically, metallic potassium can be formed at high temperature during the redox reaction between GO and KOH, causing intercalation on the graphitic type layers and, therefore, allowing the graphitic layers to split and degrade, resulting in the development of microporosity [21]. However, at low temperature, the pore development is likely incomplete, which can cause a large pore diameter. Large pores are unfavorable for CO_2_ molecule capture; instead, micropores and narrow mesopores are recommended for gas capture.

XPS analysis is sensitive to the chemical environment of elements. XPS is of paramount importance to characterize carbon-based materials. XPS was especially investigated on the activated GO, which show the best CO_2_ adsorption performances (see next section); namely GO-PA 900 and GO-PA 800. Even if XPS is not a purely quantitative technique, the atomic ratio of oxygen over carbon, O/C, from the survey scan (Supplementary Materials Figure S2) gives a rough idea of the oxygen content in the samples. Table 3 gives the O/C atomic ratio of GO, GO-PA 900 and GO-CA 800. As expected, GO shows an oxygen content that is quite high (O/C = 0.46). After both chemical and physical activation, a distinct decrease in oxygen content is observed meaning that the oxygen-containing groups (carboxyl, epoxide, or other functional groups) are decomposed by activation. The atomic percentage of oxygen was decreased from around 46 % to less than 17 % after chemical activation and down to 5 % after physical activation. As observed, depending on the reduction method and the used experimental conditions, the C/O atom ratio from XPS is not necessarily accompanied by I_D_/I_G_ decrease. It is explained by the fact that even if the nature of the functional groups, especially elimination of COOH functional groups, is modified upon the reduction reaction, the sp^2^ carbon atom hybridization (from sp^3^) is not necessarily reached. Indeed, the creation of these hydrogenated carbon atoms (C-H) or other defect types (vacancies, holes, dangling bond, etc.) does not increase the sp^2^ ratio content after reduction [30,31].

**Figure 6:**
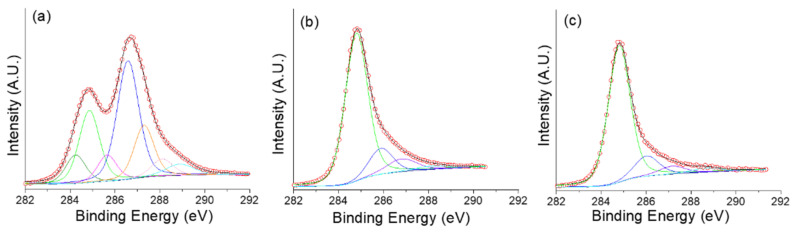
C 1s XPS spectra for (**a**) GO (**b**) GO-PA 900 and (**c**) GO-CA 800.

The C1s spectral region was analyzed for the 3 samples (Figure 6 and Table 3). The deconvoluted high resolution C1s region spectra of GO, GO-PA 900 and GO-CA 800 are presented in Figure 6. The fitting parameters including contribution assignment, Full Width at Half Maximum (FWHM) and contribution concentration are given in Table 4 and Table 5. The contribution at 284.2–284.8 eV is assigned to the sp^2^/sp^3^ carbon atoms in C=C graphene network and/or carbon atoms belonging to C-H bonds [32,33,34,35]. Higher C1s binding energy can be assigned to carbon linked to oxygen: C-O around 286 eV, C=O around 287 eV, and O=C-O at 287–289 eV. Sulfur and nitrogen containing functions observed in GO probably come from the acids used during its synthesis. This result is in agreement with O1s spectral regions shown in Supplementary Materials, Figure S3 and Tables S1–S3. After both activations, even if the main contribution peak could not be fitted with the two components namely sp^2^ and sp^3^, a broader peak is observed. The C=C/C-H contribution is observed to be significantly increased while the features assigned to oxygen linked with the carbon network are decreased for the two activated GOs. These results, in agreement with FTIR, indicate that both physical and chemical activations lead to a reduction reaction of the starting GO. Similarly, Yang et al. reported that after the reduction of GO, the intensity of all C1s peaks of hydroxyl, carbonyl, and carboxyl carbon atoms, including C-OH, decreased significantly [36]. And from XPS, it is revealed that the chemical activation approach leads to a stronger reduction.

## 4. Discussion

### Discussion and Mechanism of Activation

From the TEM analysis, a thin layer of pure GO flakes with around 5–7 layers (thickness of around 2 nm) were observed (Figure 4a,b, respectively). The smooth surface of GO was visible in the TEM pictures before activation, which is consistent with the SEM observations. The surface of activated GOs displayed a visible pore structure after both activation processes, indicating that KOH and CO_2_ may work as activators — particularly KOH forming a large number of pores.

FTIR and XPS of activated GO by both physical and chemical activation have shown a significant reduction of oxygen-containing group content. Concerning microporosity development by chemical activation, the increase in SSA appears to be significant, with an increase of a factor of 5, confirming the exceptional porous structure of the activated GO by the KOH-based method conducted at 800 °C. Additionally, from the analysis, the hysteresis loops for the sample are type H3, indicating that the pores developed are irregular and open, with good connectivity between intragranular pores with shapes of parallel, slit-like, and open-ended tubes, which are conducive for gas transport and providing space for shale gases [37]. In this study, physical activation of graphenic materials, particularly GO, is carried out using heat treatment at temperature greater than 600 °C in a partly oxidizing medium of CO_2_ and N_2_ with the main goal of maximizing internal surface area. Shen et al. further supported this, claiming that graphitic materials exhibited endothermic reactions above 700 °C, and breakdown of the carbon framework happening in this temperature range [17]. Furthermore, greater temperatures would be required for decomposition, resulting in improved chemical and physical activation efficiency [38].

**Figure 7:**
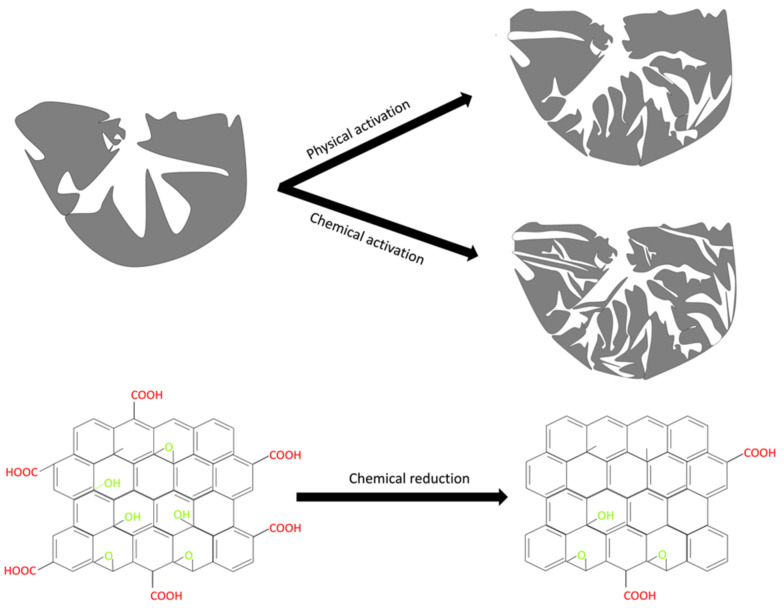
Mechanism of chemical and physical activation approaches in GO; top: modification of pore structure of GO by activation; bottom: modification of the functional groups on GO surface by activation.

Table 3 provides the information on the O/C ratios of pristine GO, GO-PA 900 and GO-CA 800. The mechanism of GO activation with CO_2_ normally involves the Boudouard reaction (Equation (1)) [39]. From the reaction, CO_2_ undergoes dissociative chemisorption on the carbon surface to create a surface oxide and form carbon monoxide (as shown in Equation (2)). Then, from Equation (3), the surface oxide, C(O), is desorbed from the surface, allowing the pore structure to develop further (as shown in Figure 7). Additionally, the CO in the gaseous product can potentially be deposited on the carbon active sites and delay the gasification process (Equation (4)).

C + CO_2_ → CO (1)

C + CO → C(O) + CO(2)

C(O) → CO(3)

C + CO → C(CO)(4)

On the other hand, after chemical activation with KOH, the morphology of the GO slowly degrades as the applied activation temperature raises and the structure of the activated GO seems to be less dense with rougher surface and many other pores, due to the activation process. First, KOH reacts with H_2_O coming from the impregnation process. After activation, the formation of pores on the GO surface can be attributed to the reaction between KOH and C in an inert atmosphere at high temperatures during the activation process, in which some species such as potassium oxide (K_2_O), potassium carbonate (K_2_CO_3_), and potassium (K) metal attempt to diffuse within the structure of the GO, thereby forming new pores and opening existing ones. The development of pores on activated GO can be explained by numerous simultaneous and sequential reactions, as shown by [27,40,41].

2KOH + 4H_2_O →K_2_O+ 5H_2_O (5)

C + 5H_2_O →CO+ 5H_2_ (6)

CO + 5H_2_O → CO_2_ + 5 H_2_ (7)

K_2_O + CO_2_ → K_2_CO_3_ (8)

K_2_O + H_2_ →2K + H_2_O (9)

K_2_O +CO →2K + CO_2_ (10)

6KOH +2C →2K +3 H_2_ +2 K_2_CO_3_ (11)

K_2_CO_3_ → K_2_O + CO_2_ (12)

K_2_CO_3_ + C → K_2_O + 2 CO_2_ (13)

K_2_O + C → 2K + CO (14)

2K + CO_2_ → K_2_O + CO (15)

Overall, in this study, it can be signified that the presence of structure defects in the adsorbent is not the sole factor that affects the CO_2_ capture capacity and the contribution of other factors such as surface area, microporosity and surface chemistry of the adsorbent should also be taken into the consideration. Indeed, we believe that activating GO at the high temperature range used for both chemical and physical approaches might lead to the development of additional porous structure.

## 5. Conclusions

In comparison to physical activation, this work demonstrates that chemical activation by KOH is more efficient for the development of high surface and porous graphene-based adsorbents. The best activation condition for this study is found to be 800 °C with 1:3 (GO: KOH ratio) and 6 h of impregnation time to obtain a porous graphene adsorbent with a highest BET surface area of 1060.5 m^2^ g^−1^ and the maximum total pore volume of 0.99 cm^3^ g^−1^. We agreed that the specific surface area, pore volume, and pore size distribution can be tuned, as increasing the activation temperature greatly improves adsorption capacity, and at high temperature, the carbon structure decomposes, resulting in the development of porous structures. Importantly, the results of the CO_2_ adsorption study clearly demonstrates that lower acidity (higher reduction level) of highly porous graphene with high surface area and volume, as well as further pore size reduction, play a vital role in achieving higher CO_2_ adsorption capacities. Aside from these factors, we believe that the surface chemistry i.e., the nature of the functional groups may have an impact on the adsorbent capacity of carbon-based materials, particularly graphene. Indeed, functional groups especially nitrogen-containing groups, heteroatom doping, metal or metal oxide doping, and organic polymer surface coating may promote an increase in functional basicity, which has been proved to enhance adsorption capacity.

## Figures and Tables

**Table 1:** CO_2_ adsorption capacity of pristine and activated GO via different activation approaches.

Approach	Sample	Adsorption Capacity (mg g^−1^)	Reference
Physical activation	GO	4.2	This study
	GO-PA 500	9.6	
	GO-PA 600	10.2	
	GO-PA 700	12.3	
	GO-PA 800	15.3	
	GO-PA 900	27.2	
Chemical activation	GO	4.3	
	GO-CA 500	10.8	
	GO-CA 600	25.0	
	GO-CA 700	32.8	
	GO-CA 800	38.9	

**Table 2:** Surface and porosity analysis of GO and the activated GO.

Sample	S_BET_ (m^2^ g^−1^)	Total Pore Volume (cm^3^ g^−1^)	Micropore Volume (cm^3^ g^−1^)	Microporosity Fraction (%)	Pore Size (nm)
GO	219.3	0.79	0.007	0.88	13.67
GO-PA 900	762.5	2.31	0.039	1.69	12.14
GO- CA 800	1060.5	0.99	0.131	13.19	3.65

**Table 3:** O/C atomic ratio from XPS survey spectra of GO, GO-PA 900 and GO-CA 800.

Sample	O/C (Atomic Ratio)
GO	0.46
GO-PA 900	0.05
GO-CA 800	0.17

**Table 4:** Decomposition of C1s features of GO.

	C=C	C-C	C-N	C-O	C-S2	O=C-N	O=C-O
Position (eV)	284.25	284.86	285.65	286.58	287.30	288.10	288.89
FWHM	1.04	1.07	1.01	1.14	1.04	1.12	1.49
Concentration (%)	8.27	21.54	7.31	37.75	15.11	5.37	4.65

**Table 5:** Decomposition of C1s features of GO-PA 900 and GO-CA 800.

**GO-PA 900**	**C-C**	**C-O**	**C=O**	**O=C-O**
Position (eV)	284.81	285.88	286.69	287.93
FWHM	1.18	1.11	1.37	1.63
Concentration (%)	75.57	12.37	8.11	3.95
**GO-CA 800**	**C-C**	**C-O**	**C=O**	**O=C-O**
Position (eV)	284.80	286.02	287.16	288.65
FWHM	1.15	1.28	1.38	1.50
Concentration (%)	78.65	12.92	5.44	2.99

## Data Availability

Not applicable.

## Supplementary Materials

The following are available online at https://www.mdpi.com/article/10.3390/nano11092419/s1, Figure S1: Interspacing Distance of GO from TEM; Figure S2: Wide range XPS spectra of GO, GO-PA 900 and GO-CA 800; Figure S3: XPS O1s Region of GO, GO-PA 900 and GO-CA 800 and Fitting Parameters; Table S1: Fitting parameters and concentration of the O1s signal of GO; Table S2: Fitting parameters and concentration of the O1s signal of GO-PA 900; Table S3. Fitting parameters and concentration of the O1s signal of GO-CA 800.
