# Supplementary material for: Physical and Chemical Activation of Graphene-Derived Porous Nanomaterials for Post-Combustion Carbon Dioxide Capture
Source: Nanomaterials (Basel). 2021 Sep 17;11(9):2419. doi: 10.3390/nano11092419 (PMC8466215; doi:10.3390/nano11092419)
Supplement: Supplementary file 1 [file nanomaterials-11-02419-s001.zip › nanomaterials-1323137-supplementary.pdf]

nanomaterials

MDPI

Supplementary Materials

Physical and Chemical Activation of Graphene-Derived Porous Nanomaterials for Post-Combustion Carbon Dioxide Capture

Rabita Mohd Firdaus [1,2], Alexandre Desforges [2], Mélanie Emo [2], Abdul Rahman Mohamed [1,*] and Brigitte Vigolo [2,*]

1 School of Chemical Engineering, Engineering Campus, Universiti Sains Malaysia, Nibong Tebal 14300, Malaysia; rabitafirdaus58@student.usm.my
2 Université de Lorraine, CNRS, IJL, F-54000 Nancy, France; Alexandre.Desforges@univ-lorraine.fr (A.D.); Melanie.Emo@univ-lorraine.fr (M.E.)
* Correspondence: chrahman@usm.my (A.R.M.); Brigitte.Vigolo@univ-lorraine.fr (B.V.); Tel.: +604-599-6410 (A.R.M.); Tel.: +33-372-742594 (B.V.)

1. Interspacing Distance of GO from TEM

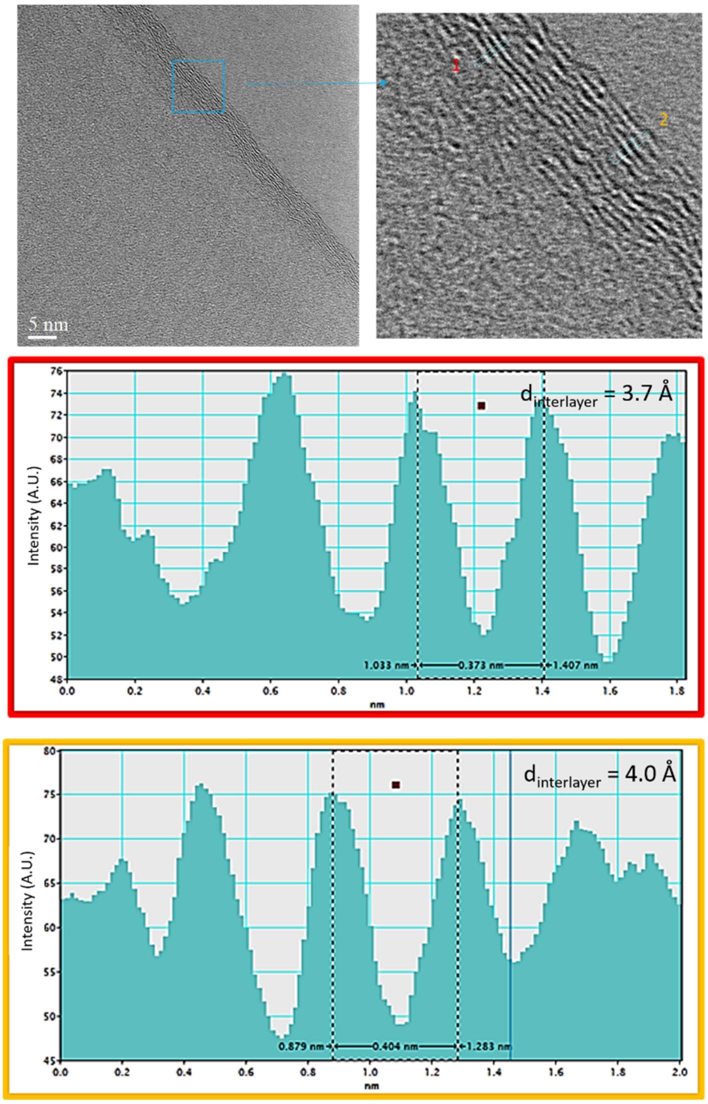

Figure S1. Measurement of interspacing layer of GO from TEM.

2. XPS Survey Scans of GO, GO-PA 900 and GO-CA 800

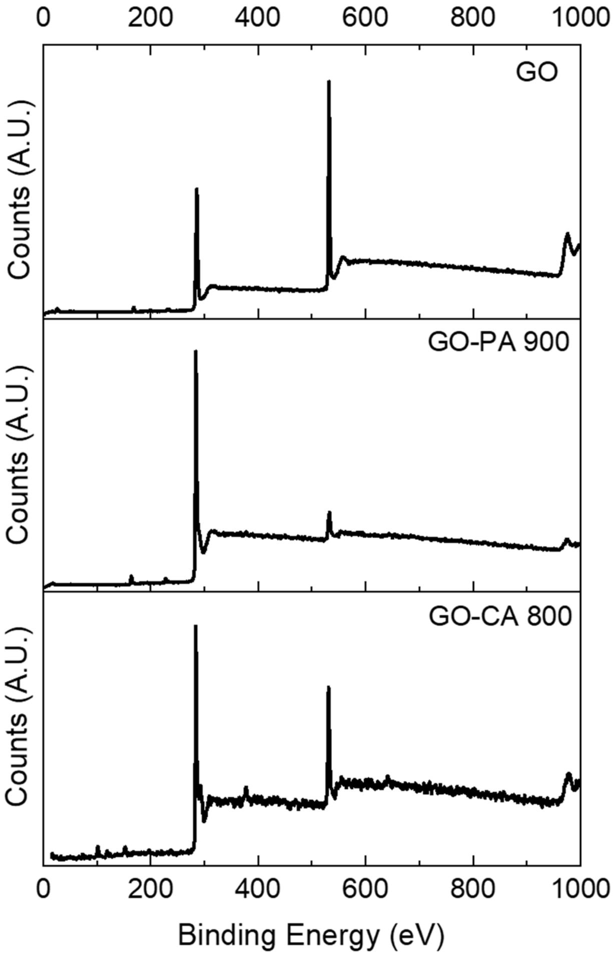

Figure S2. Wide range XPS spectra of GO, GO-PA 900 and GO-CA 800.

3. XPS O1s Region of GO, GO-PA 900 and GO-CA 800 and Fitting Parameters.

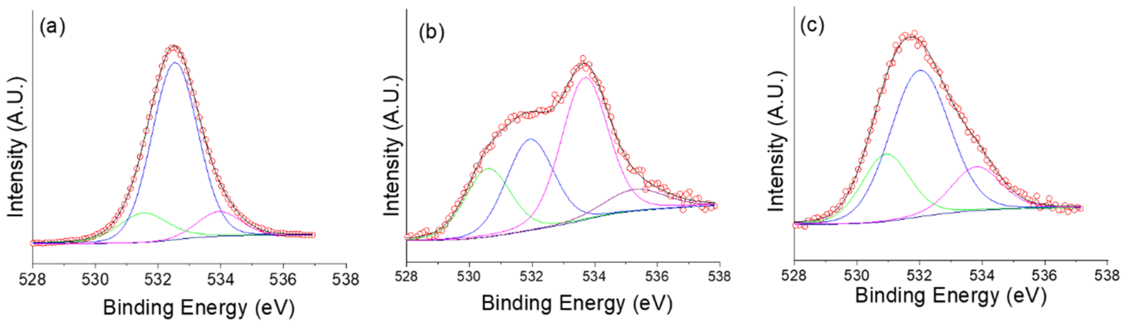

Figure S3. XPS O1s region of (**a**) GO, (**b**) GO-PA 900 and (**c**) GO-CA 800.

Table S1. Fitting parameters and concentration of the O1s signal of GO.

	**O=C-N**	**O-C**	**O=C-N**
Position (eV)	531.52	532.53	533.94
FWHM	1.86	1.72	1.61
Concentration (%)	10.79	79.64	9.57

Table S2. Fitting parameters and concentration of the O1s signal of GO-PA 900.

	**O=C**	**O-C**	**O3S**	**O-C=O**
Position (eV)	530.59	531.93	533.68	535.29
FWHM	1.72	1.77	1.76	2.26
Concentration (%)	20.49	27.99	43.07	8.45

Table S3. Fitting parameters and concentration of the O1s signal of GO-CA 800.

	**O=C**	**O-C**	**O-C=O**
Position (eV)	530.92	532	533.81
FWHM	1.80	2.23	1.86
Concentration (%)	23.90	60.89	15.21

© 2021 by the authors. Licensee MDPI, Basel, Switzerland. This article is an open access article distributed under the terms and conditions of the Creative Commons Attribution (CC BY) license (http://creativecommons.org/licenses/by/4.0/).
